# Norepinephrine as an Enhancer Promoting Corneal Penetration of Riboflavin for Transepithelial Corneal Crosslinking

**DOI:** 10.1167/tvst.12.2.21

**Published:** 2023-02-14

**Authors:** Guoying Liu, Tan Li, Benxiang Qi, Ganyu Gong, Tengyou Guo, Qingjun Zhou, Vishal Jhanji, Bi Ning Zhang, Xianli Du

**Affiliations:** 1 Medical College, Qingdao University, Qingdao, China; 2 Eye Institute of Shandong First Medical University, Qingdao Eye Hospital of Shandong First Medical University, Qingdao, China; 3 State Key Laboratory Cultivation Base, Shandong Provincial Key Laboratory of Ophthalmology, Qingdao, China; 4 School of Ophthalmology, Shandong First Medical University, Qingdao, China; 5 Department of Ophthalmology, University of Pittsburgh School of Medicine, Pittsburgh, PA, USA

**Keywords:** norepinephrine (NE), enhancer, riboflavin, transepithelial corneal collagen crosslinking (CXL)

## Abstract

**Purpose:**

Previously, we found norepinephrine (NE) could affect the corneal epithelial integrity, herein we investigated the feasibility and safety of NE serving as a chemical enhancer to promote corneal penetration of riboflavin during transepithelial corneal crosslinking (CXL).

**Methods:**

The dosage of NE that could promote riboflavin diffusion through the healthy epithelial barrier without inducing epithelial damage in C57BL/6 mice was determined. The safety of NE treatment was confirmed by morphological and histological examinations of the whole cornea. The efficacy of NE in promoting riboflavin penetration was verified by slit lamp and scanning electron microscope (SEM), and corneal biomechanical measurement after CXL. To better fit the clinical scenario, increased NE dosage and shortened riboflavin infiltration time were further evaluated.

**Results:**

The lowest dosage of NE (1 mg/mL) that facilitated transepithelial riboflavin permeation was 2 µL. No visible corneal structure alteration was observed after NE treatment. SEM indicated dissociation of intercellular junctions among corneal epithelial cells. Homogenous distribution of riboflavin throughout corneal stroma was observed. NE-treated corneas reached comparable biomechanical properties after CXL, including stress-relaxation curve and elastic modulus, with corneas treated with the commercially available transepithelial drug Peschke TE. To better fit the clinical scenario, increasing NE up to 5.5 µL helped riboflavin infiltrate the corneal stroma within 30 minutes. After CXL with 9 mW/cm^2^ ultraviolet-A (UVA) for 2.5 minutes, the cornea showed significantly enhanced corneal biomechanical properties with undisturbed corneal endothelium.

**Conclusions:**

NE serves as an effective enhancer in increasing riboflavin diffusion with limited impairment on corneal epithelium and has great potential for clinical application.

**Translation Relevance:**

NE serves as an effective enhancer for riboflavin penetration and clinical transepithelial CXL.

## Introduction

Keratoconus (KC) is an ocular disorder characterized by progressive corneal thinning, corneal steepening, and asymmetric corneal ectasia leading to visual impairment.^1^ Corneal collagen crosslinking (CXL) has been approved for the management of progressive KC. The combination of riboflavin and ultraviolet-A (UVA; 365 nm) strengthens the corneal biomechanical profile. Although widely applied in the clinic, CXL protocols are being adjusted repeatedly to improve riboflavin penetration^2^^–^^4^ and UVA irradiation regimen,^5^^–^^8^ to achieve better therapeutic effects and post-surgical outcomes.

Standard CXL (epi-off) and transepithelial (epi-on) CXL protocols are two major CXL surgical strategies. Standard CXL has better therapeutic effects owing to better penetration of riboflavin into the corneal stroma by removing the corneal epithelium, but it has been associated with corneal pain, infectious keratitis, and abnormal epithelial wound-healing.^9^^,^^10^ The transepithelial CXL procedure relies on chemical enhancers, such as benzalkonium chloride and ethylenediaminetetraacetic acid, to load riboflavin into the corneal stroma without removing the epithelium, which is particularly appealing for young patients and patients with thinner corneas.^11^^,^^12^ Overall, the riboflavin infiltration efficiency via chemical enhancers in transepithelial CXL is lower compared with epithelial removal in standard CXL, due to the strong barrier function of the corneal epithelium.^13^ Moreover, these chemical enhancers, like benzalkonium chloride, are toxic to the ocular surface.^14^^–^^16^ Hence, it is necessary to explore safer and more effective CXL enhancers.

The corneal epithelium serves as a strong barrier separating the environment and the intraocular space. The corneal epithelial barrier is composed of tight junctions and adhesive junctions, which not only effectively blocks pathogenic microorganism invasion but also blocks topically applied drug infiltration.^17^ The opening of epithelial junctions could facilitate the penetration of riboflavin. Norepinephrine (NE) is a classical stress hormone released by sympathetic nerves.^18^ Sympathetic nerves are widely spread in the ocular anterior segment, projecting to the ciliary body, iris sphincter pupillae muscle, lacrimal gland, and limbus.^19^^,^^20^ Sympathetic neurotransmitter NE has been reported to weaken colonic barrier via decreasing tight junction expression.^21^ Therefore, we hypothesized that supplementing low-dose NE to the ocular surface could allow riboflavin to overcome the epithelial barrier and penetrate the stroma. This study aimed to investigate the safety and efficacy of low-dose NE as an enhancer for transepithelial CXL. Moreover, we also explored a clinical CXL protocol by increasing the dosage of NE and decreasing the riboflavin penetrating duration to further verify its clinical applicability.

## Materials and Methods

### Animal Ethics

Seven to 10-week-old C57BL/6 mice of both genders (Vital River Laboratory Animal Technology Co., Ltd., Beijing, China) were used for this study. All animal experiments were carried out in accordance with the standards in the ARVO Statement for the Use of Laboratory Animals in Ophthalmic and Vision Research.

### NE Treatment

Anesthetization was conducted by intraperitoneal administration of 0.6% pentobarbital sodium. A gradient dose of NE ((R)-(-)-norepinephrine L-bitartrate monohydrate; Grand Pharma, Wuhan, China) at the concentration of 1 mg/mL was subconjunctivally injected into mice. The optimal dosage was determined to allow effective riboflavin penetration with minimal corneal damage. After the lowest efficient dosage of NE was determined, the same dosage of NE (2 µL) was applied to the right eye of each mouse by subconjunctival injection, and an equal amount of NaCl solution was applied to the left eye as a control. To better suit the clinical scenario, an increased amount to 5.5 µL NE was utilized to the simulate corneal collagen crosslinking scheme (S-CXL group). In the S-CXL experiments, riboflavin was allowed for a 30-minute penetration into the stroma, which was close to the clinical penetrating time.

### Anterior Segment Assessment

Corneal status was examined at 3, 8, 15, and 24 hours after NE treatment. Slit lamp examination and anterior segment optical coherence tomography (OCT; RTVue-100; Optovue, Fremont, CA, USA) were used to examine the corneal morphology and riboflavin infiltration status.

### Scanning Electron Microscopy Analysis

Corneal epithelial cell tight junctions were examined with scanning electron microscopy (SEM). Briefly, corneal buttons were collected and fixed in 2.5% glutaraldehyde, and then washed with 0.1 M phosphate-buffered saline (PBS) buffer solution 3 times. Samples were dehydrated by immersion into 30%, 50%, 70%, 80%, and 100% ethanol and then isoamyl acetate. Samples were dried and mounted on SEM stubs using carbon adhesive tabs. Samples were then sputter-coated with a 10 nm thick layer of gold (Bal-Tec) and examined with a scanning electron microscope (JSM-840; JEOL, Tokyo, Japan).

### In Vitro Immunostaining of Cellular Tight Junctions

Human corneal epithelial cells (HCECs; ATCC) were cultured to full confluence on a 24-well plate with sterile glass coverslips at the bottom. After being starved by DF12 supplemented with 0.1% fetal bovine serum (FBS) for 1 day, cells were treated with corresponding drugs. Either 3.4 µL 10 µM NE or an equal amount of PBS as control were added to each well for 1 hour and then replaced with fresh culture medium for another 2.5 hours before cells were collected for immunostaining. Cells were fixed with methanol for 20 minutes at −20°C, washed 3 times with PBS, and then stained with ZO-1 antibody (Invitrogen; 40-2200, 1:200) overnight at 4°C. After rewarming for 30 minutes, samples were washed 3 times with PBS, subsequently incubated in donkey anti-rabbit IgG H&L (Alexa Fluor 594, Abcam, ab150076; 1:500) for 1.5 hours, dyed with DAPI (Solarbio; c0065), and examined by fluorescence microscopy. This experiment was performed for three times, with one treatment and one control group each time.

### Hematoxylin and Eosin Staining

Corneas were fixed with 4% paraformaldehyde (PFA) and embedded in paraffin. Then, 5 µm thick sections were made. Sections were dried for 3 hours and deparaffinized in xylene. Sections were rehydrated and stained with hematoxylin and eosin.

### Corneal Endothelial Alizarin Red Staining

Corneas were harvested and immersed in 1% Alizarin Red solution for 80 seconds. After washing twice with saline solution, corneas were laid flat on a glass slide and imaged under a bright field with the Eclipse TE2000-U microscope (Nikon, Tokyo, Japan). Endothelial cell density was analyzed with a noncontact specular microscope (Konan Medical, Inc.).

### Riboflavin Penetration and Examination

Isotonic riboflavin solution (0.06%) was prepared with riboflavin sodium phosphate powder (Taisheng Pharmaceutical Company, China) dissolved in 0.9% saline. A hydraulic column was made to mimic the corneal silicon ring used during the clinical CXL surgery, to facilitate riboflavin penetration by making a liquid column pressure on the ocular surface. Briefly, the hydraulic column was gently fixed along the palpebral margin with the column edge being 0.15 cm from the corneal edge. Riboflavin was filled into the column, making a liquid column with a height of 1.5 cm. To examine the homogeneity of intrastromal penetrated riboflavin, corneas were collected for 3D scanning with a confocal microscope (Zeiss, Oberkochen, Germany) after riboflavin infiltration. Two mm central corneal buttons were dissected and flat mounted on a glass slide for confocal scanning. The depth and uniformity of intrastromal riboflavin were documented.

For the 2 µL NE treatment group, mice were anesthetized at 15 hours after NE application, and riboflavin was allowed to penetrate for around 2 to 3 hours. For the 5.5 µL NE treatment S-CXL group, mice were anesthetized at 8 hours after NE application, and riboflavin was allowed to penetrate for around 30 minutes. Peschke TE (Peschke Trade GmbH, Huenenberg, Switzerland) is the commercially available transepithelial CXL drug currently used in the clinic. Peschke TE was administered directly on the cornea for 30 minutes as the positive control.

### Corneal Collagen Crosslinking

A corneal area with 4 mm diameter was illuminated by a UVA LED light (M365LP1; Thorlabs, Newton, NJ, USA), with the emission wavelength being 365 nm. The energy was monitored by a UVA Radiometer (Uvata, Shanghai, China). We applied the standard CXL irradiation parameters commonly used in human CXL surgery in our study, which is 3 mW/cm^2^ UVA light for 30 minutes, 5.4 J/cm^2^ of cumulative fluence. Both the 2 µL NE treatment group and the Peschke TE were crosslinked under this setting.

We further adopted an optimized CXL protocol in the S-CXL group, which was 9 mW/cm^2^ for 2.5 minutes and a surface dose of 1.53 J/cm^2^.^22^ After CXL, Tobramycin and Dexamethasone Eye Ointment (TobraDex; Alcon, Vilvoorde, Belgium) was applied to help corneal recovery in the S-CXL group.

### Indentation Test

Indentations of different regions of the ocular surface, including the corneal stroma, limbal region, and sclera, had been conducted.^23^ In our study, we applied this technique optimally to measure the corneal mechanical properties of mice post-CXL. Briefly, immediately after euthanizing mice by neck amputation, the post-CXL eyeballs were collected and embedded in Tissue-Tek O.C.T. Compound (Sakura Finetek, Alphen aan den Rijn, Netherlands), and shock-frozen at −80°C refrigeration. Sagittal sample sections with a thickness of 100 µm were prepared by using a Leica CM1950 cryostat (Leica, Germany). The sections were placed on adhesion microscope slides (Citotest, China) and fixed at the bottom of the culture dishes. Samples were thawed and equilibrated to room temperature. Samples were immersed in PBS during the entire indentation test.

The mechanical properties of the corneal stroma were measured by a Piuma Nanoindenter system (OPTICS11, Amsterdam, The Netherlands). A spherical tipped indenter with a radius of 24.5 µm and a cantilever with a stiffness of 0.59 N/m were used for all indentation tests. Indentation points located in the middle of each specimen were loaded. For a stress-relaxation test, a 20 µm depth of indentation was performed with a period of 1 second, and the maximum indentation depth was kept for 300 seconds before unloading. Moreover, a quasi-static indentation test was applied to measure the elastic modulus of cornea by performing a 10 µm depth of indentation with a period of 2 seconds. All results were calculated as the average of three indentations.

Finally, the mechanical data were analyzed. The stress-relaxation curves were fitted with the third order Prony series model, writing as follows:

$$\begin{array}{c} G\left(t\right)=1-\sum_{j=1}^{3}a_{j}\left(1-e^{-t/\tau_{j}}\right). \end{array}$$

Specially, let *G* (∞) be t→∞ lim G(t), with it is equal to (1 − *a*_1_ − *a*_2_ − *a*_3_) and τ_1_, τ_2 _, and τ_3_ are relaxation time constants. The Hertz function was used to automatically fit the indentation curve to obtain the elastic modulus of cornea on the indentation position.

### Statistical Analysis

Each experiment was repeated at least three times. Student's *t*-test was applied to analyze the difference between the NE and the Peschke TE groups and the difference between the S-CXL and healthy groups with GraphPad Prism 8.0 (GraphPad Software, La Jolla, CA, USA). The data were presented as mean ± standard error of the mean. *P* < 0.05 indicated a significant difference.

## Results

### Safety of Low Dose NE on the Ocular Surface

**Figure 1.**
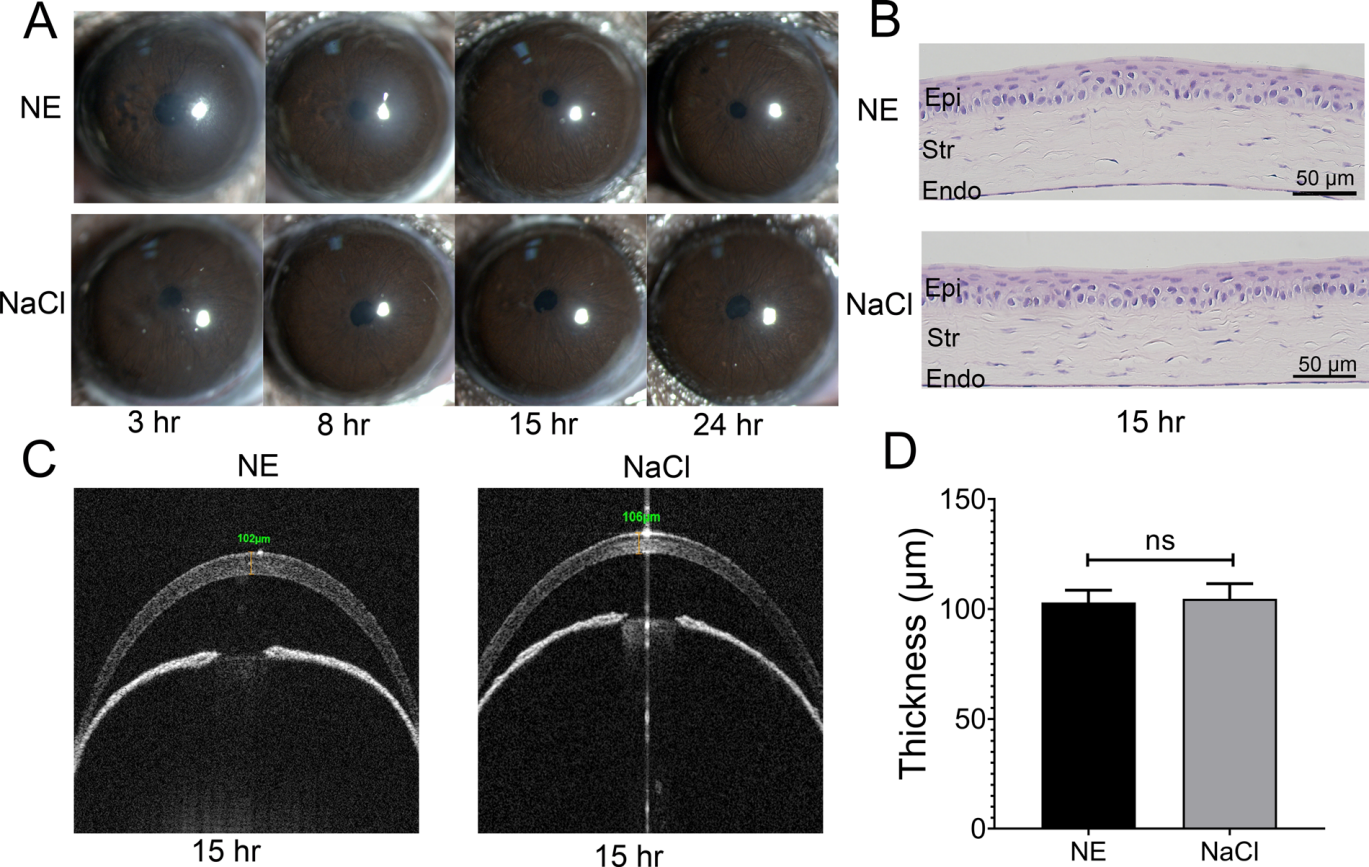
**Safety examinations of low-dose NE application to the ocular surface.** (**A**) Representative silt lamp images at 3, 8, 15, and 24 hours after subconjunctival injection of NE and NaCl, respectively. Slight edema was observed at the early stage after NE application and the cornea turned transparent at 15 hours after NE treatment (*n* = 3). (**B**) No inflammatory infiltration and structural alteration after NE treatment was observed in the histological sections at 15 hours (*n* = 3). (**C**) OCT indicated there was no difference in corneal structure and thickness between the NE group and the control group at 15 hours (*n* = 3). (**D**) The central corneal thickness was 104.6 ± 6.9 µm after NE treatment and 102.9 ± 5.6 µm in the NaCl group. There was no statistical difference (*n* = 11 and 13, respectively).

Then, 2 µL, 2.5 µL, and 3 µL NE were applied by subconjunctival injection and all of these doses could promote riboflavin infiltration. Although NE is a natural neurotransmitter, we selected the lowest dose to minimize potential adverse effects. Corneal histological examination and OCT examination at 3 hours and 8 hours after NE treatment showed an increased corneal thickness compared control group (Supplementary Fig. S1A, S1B). Corneas recovered to full transparency at 15 hours after 2 µL NE treatment (Fig. 1A), and there was no structural difference between the treated cornea and the control at this time point, with no inflammation observable (Fig. 1B). Consequently, the corneal thickness was also returned to normal at 15 hours according to histology and OCT (see Fig. 1C, Supplementary Fig. S1A, S1B). The central corneal thickness was 104.6 ± 6.9 µm in the 2 µL NE treatment group and was 102.9 ± 5.6 µm in the NaCl group, without significant difference (*P* = 0.5094; Fig. 1D). All these results indicated that the application of 2 µL NE to the ocular surface was safe and no obvious complication could be observed.

### Dissociation of Corneal Epithelial Junctions After NE Treatment

**Figure 2.**
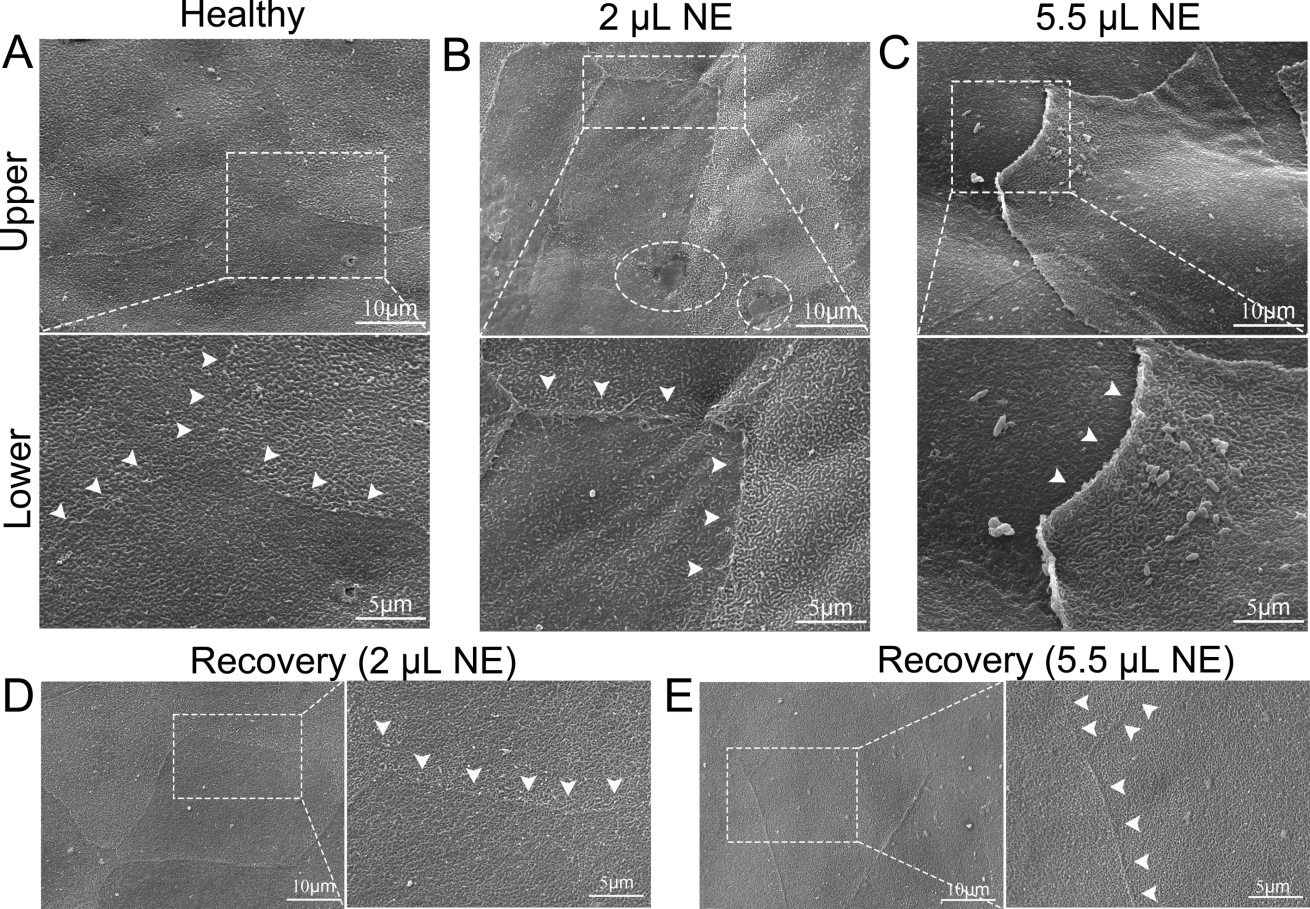
**Dissociation and recovery of corneal epithelial junctions after NE treatment.** The *lower panel* images in **A**, **B**, and **C** are the higher magnification of selected areas in the *upper panel*. The *right panel* images in **D** and **E** are the higher magnification of selected areas in the *left panel*. (**A**) Healthy polygonal corneal epithelium cells are closely linked to each other with the intercellular junctions (*arrowheads*) barely visible (*n* = 3). (**B**) Widened intercellular junctions could be spotted (*arrowheads*), and the cellular boundaries raised slightly like a ridge with potential dissociation at the junctions after 2 µL NE treatment at 15 hours. In addition, the possible dissociation of cellular joint points was observed (*circles*; *n* = 3). (**C**) Partial lifting at the edges of cells with more obvious cellular boundaries (*arrowheads*) was observed, indicating the dissociation of the epithelial cells and the disruption of the barrier integrity at 8 hours after high dose NE treatment (*n* = 3). (**D,** **E**) The epithelial integrity was fully recovered in both the 2 µL and 5.5 µL NE treatment groups in the following 5 to 7 days. There is no observable difference between the NE-treated corneal epithelium and the healthy corneal epithelium (*n* = 4 in the 2 µL NE group and *n* = 3 in the 5.5 µL NE group).

To validate the efficacy of NE on the disruption of the epithelial barrier, we examined epithelial junctions with SEM. As was shown in Figure 2A, the healthy corneal epithelium has polygonal cells closely linked to the adjacent cells with the intercellular junctions barely visible. After 2 µL NE treatment, widened intercellular junctions could be spotted, and the cellular boundaries raised slightly like a ridge with potential dissociation at the junctions (Fig. 2B). In order to achieve greater dissociation of epithelial intercellular junctions, we further applied NE up to 5.5 µL and examined the intercellular junctions. Partial lifting at the edges of the cells with more obvious cellular boundaries was observed (Fig. 2C), indicating the dissociation of the epithelial cells and the disruption of the barrier integrity after high-dose NE treatment. Moreover, in the in vitro cell culture experiment, when treated with NE, the human corneal epithelial cells displayed dissociation of tight junction marker zonula occludens-1 (ZO-1; Supplementary Fig. S2), indicating NE indeed could disrupt epithelial barrier. Nevertheless, when followed up for 5 to 7 days, the epithelial integrity was fully recovered in both the 2 µL and 5.5 µL NE treatment groups (Figs. 2D, 2E). There is no observable difference between the NE-treated corneal epithelium and the healthy cornea under SEM (see Figs. 2D, 2E).

### Low-Dose NE Promoted Riboflavin Penetration

Having confirmed that low dose NE (2 µL) could loosen the intercellular junctions, we applied riboflavin on the ocular surface to explore whether NE could serve as an enhancer for riboflavin penetration. To set up positive controls for intraocular riboflavin localization, we directly injected riboflavin into the anterior chamber and the stroma, respectively. After the intracameral injection, diffuse green fluorescence was observed between the iris and the cornea under the slit lamp white light illumination. The anterior chamber was full of riboflavin after intracameral injection, with a visible intense green stripe but an invisible iris blue line under cobalt blue light illumination (Supplementary Fig. S3A). The green stripe stands for the reflection of riboflavin and the iris blue line was supposed to be the reflection of the iris under cobalt blue light illumination. Whereas in the intrastromal injection group, riboflavin was mainly concentrated inside the cornea. A green stripe and an iris blue line were both visible under the cobalt blue light illumination (see Supplementary Fig. S3B). As the green stripe was the reflection of riboflavin, its position was in between the cornea and the iris in both intracameral and intrastromal injection groups. The intensity of the green stripe in the intrastromal injection group was weaker than in the intracameral injection group. Both the intensity of the green stripe and the existence of the iris blue line were applied as the major differences between the intracameral and intrastromal injection groups. If riboflavin reaches into the anterior chamber, the iris reflection will be blocked by the dense green reflection, leading to the disappearance of the iris blue line. If riboflavin only reaches the cornea, the iris blue line can be seen under cobalt blue light. No green stripe was observed under the cobalt blue light without injection of riboflavin (see Supplementary Fig. S3C). A corneal blue line was regarded as the reflection of the cornea under cobalt blue light illumination. These positive and negative controls could be used to indicate the riboflavin infiltration status after NE treatment.

**Figure 3.**
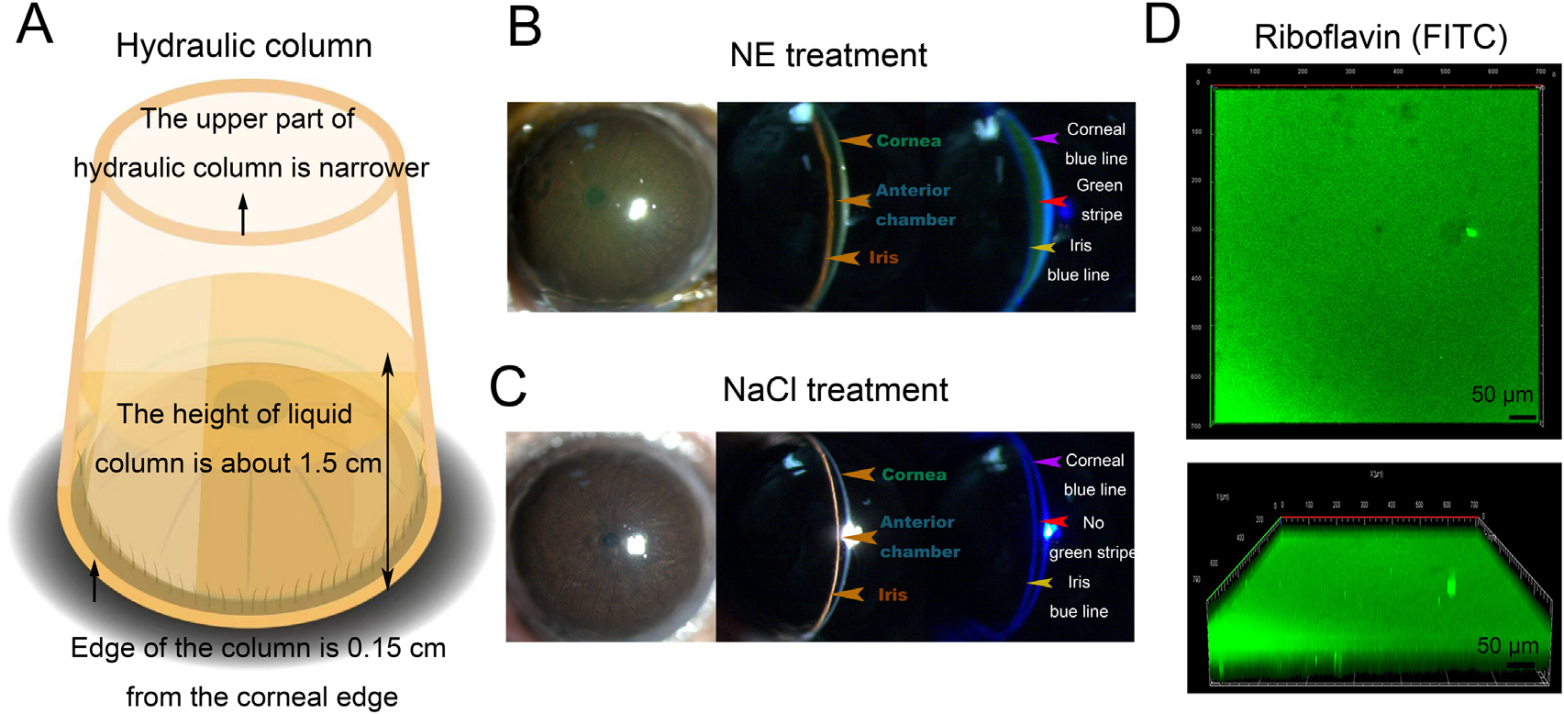
**NE mediated efficient riboflavin penetration inside the cornea.** (**A**) A schematic diagram of the hydraulic column used in our study with detailed parameters indicated. (**B**) Slit lamp examinations of the cornea after NE treatment and riboflavin penetration under the white light (*left* and *middle* photographs) and the cobalt blue light (*right* photograph). Homogeneous green fluorescence was observed all over the corneal region (*middle* photograph). The green stripe (*red arrowhead*) with a visible iris blue line (*yellow arrowhead*) indicated the riboflavin penetrated through the corneal stroma. *Purple arrowhead*: Corneal blue line (n = 3). (**C**) Slit lamp images of the cornea after NaCl treatment and riboflavin penetration under the white light (*left* and *middle* photographs) and cobalt blue light (*right* photograph). No green fluorescence was observed in the cornea (*middle* photograph) and no green stripe (*red arrowhead*) could be observed (*n* = 3). (**D**) Three-D scanning confocal microscopy of the NE-treated cornea to examine riboflavin distribution. Uniform green signals were detected (*n* = 3).

To guarantee a homogenous diffusion of riboflavin inside the cornea, we applied a hydraulic column mimicking the ring used during clinical CXL surgeries, with riboflavin filled inside the column to create hydraulic pressure on the ocular surface. The hydraulic column was gently fixed along the palpebral margin with the column edge being 0.15 cm from the corneal edge. The upper part of the hydraulic column was narrower than the lower part. Riboflavin was filled into the column, making a liquid column with a height of around 1.5 cm (Fig. 3A). The riboflavin column was set at 15 hours after 2 µL NE treatment and riboflavin was allowed to diffuse for 2 to 3 hours. Homogeneous green fluorescence could be observed all over the corneal region and both the green stripe and an iris blue line were visible (Fig. 3B), similar to the intrastromal injection cornea (see Supplementary Fig. S3B), indicating that riboflavin had penetrated through the stroma. By comparison, there was no green fluorescence and no green stripe in the NaCl-treated eye (Fig. 3C). To examine whether the riboflavin diffused homogenously inside the cornea, we collected the corneal button after penetration and performed 3D scanning with a confocal microscope. The whole button presented strong green fluorescence both vertically and laterally (Fig. 3D). In summary, low dose NE could effectively promote riboflavin penetration into the cornea and the hydraulic column helped homogeneous diffusion.

### Comparison of Efficacy Between NE and the Commercially Available Transepithelial Drug

**Figure 4.**
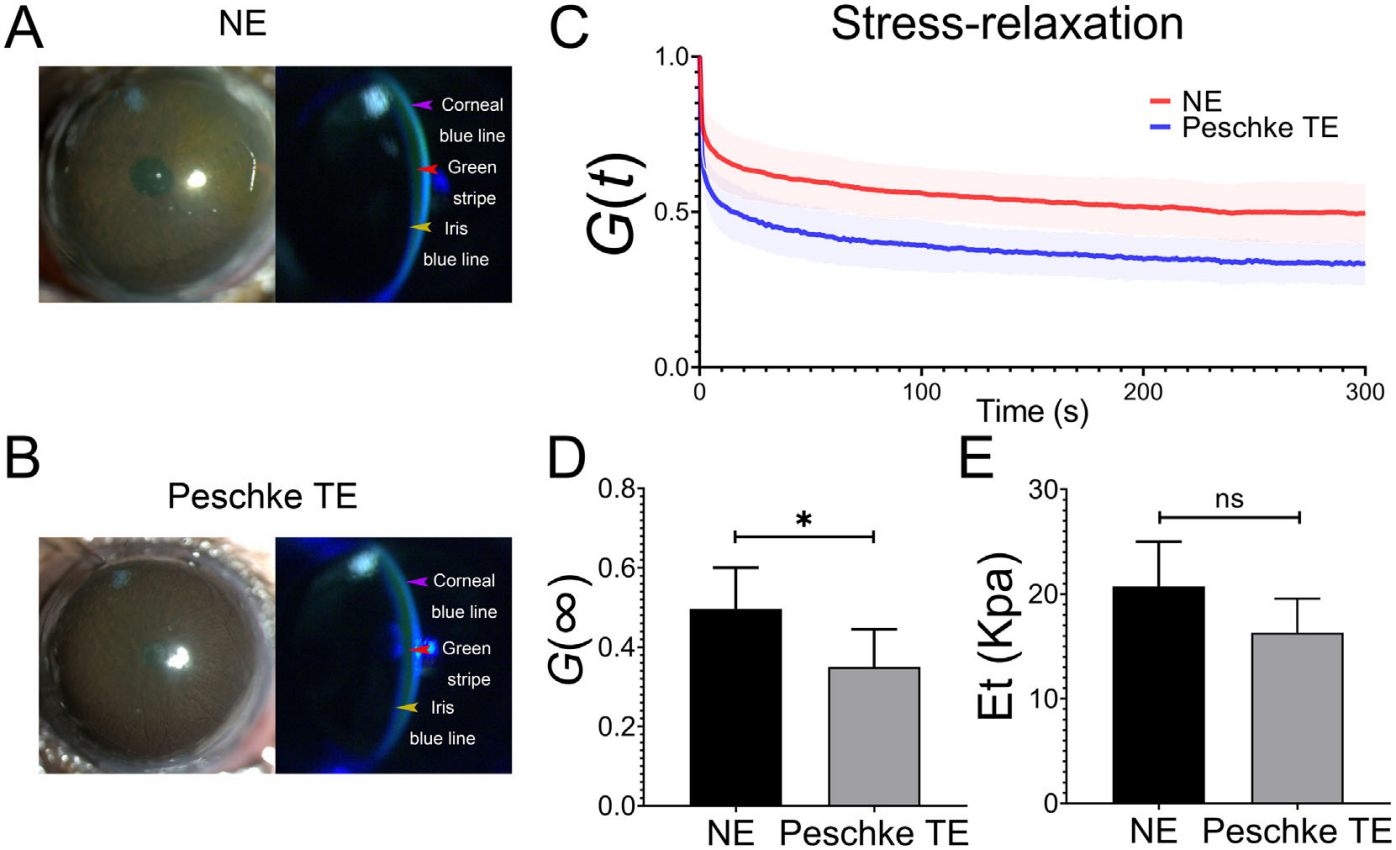
**NE-treated cornea displayed comparable CXL effects with Peschke TE.** (**A**) Slit lamp images of the riboflavin diffusion. Riboflavin was allowed to penetrate for 2 to 3 hours after 15 hours of NE treatment. A green stripe (*red arrowhead*) was visible due to the yellowish green fluorescence spreading throughout the cornea. The iris blue line (*yellow arrowhead*) was visible as the reflection from the iris under the blue cobalt light, indicated the riboflavin diffused into deep cornea. *Purple arrowhead*: Corneal blue line (*n* = 3). (**B**) Slit lamp images of the riboflavin diffusion after treatment with Peschke TE for 30 minutes. Both the green stripe (*red arrowhead*) and an iris blue line (*yellow arrowhead*) indicated the riboflavin diffused into the cornea. *Purple arrowhead*: Corneal blue line (*n* = 3). (**C**) Normalized stress-relaxation curves of NE and Peschke TE treatment groups after CXL. The *line* stands for the average value and the *shadow* indicates the standard deviation (*n* =7 and 5, respectively). (**D**) The NE group had a larger normalized stress relaxation limit *G* (∞) than the Peschke TE group (*n* = 7 and 5, respectively). *P < 0.05. (**E**) No significant difference in elastic modulus was observed between the NE and Peschke TE groups (*n* = 7 and 5, respectively).

To access the potential of NE as a transepithelial enhancer during CXL, we compared the efficacy of NE and a commercially available transepithelial drug, Peschke TE, in promoting riboflavin penetration in mice corneas. Initially, we applied the same riboflavin infiltration protocol on both groups for easy comparison. However, Peschke TE caused severe corneal damage after 3 hours infiltration (Supplementary Fig. S4A), whereas NE-treated epithelium stayed in a relatively healthy status (see Supplementary Fig. S4B). We then shortened the riboflavin application time to a close clinical typical application time, that is 30 minutes, in the Peschke TE group, to assure there was no observable corneal damage. Both the green stripe and the iris blue line were visible after treatment of both NE and Peschke TE, indicating the riboflavin had penetrated into the corneal stroma in both groups (Figs. 4A, 4B). As riboflavin reached the corneal stroma, we subjected the mice to CXL.

The operation of the indentation test was demonstrated by the presentation of the operating interface (Supplementary Fig. S5). Indentation test results showed that the velocity of stress relaxation of NE treatment group was lower than that of the Peschke TE treatment group (Fig. 4C), corresponding to which, *G* (∞) of NE treatment group was significantly increased than that of the Peschke TE treatment group (Fig. 4D). There was no statistically significant difference between results of the corneal elastic modulus of the NE and Peschke TE treatment groups (Fig. 4E). These encouraging results demonstrated that an NE-treated cornea could have comparable CXL effects with Peschke TE.

### Riboflavin Delivered by NE is Feasible for Crosslinking Under Clinical Settings

**Figure 5.**
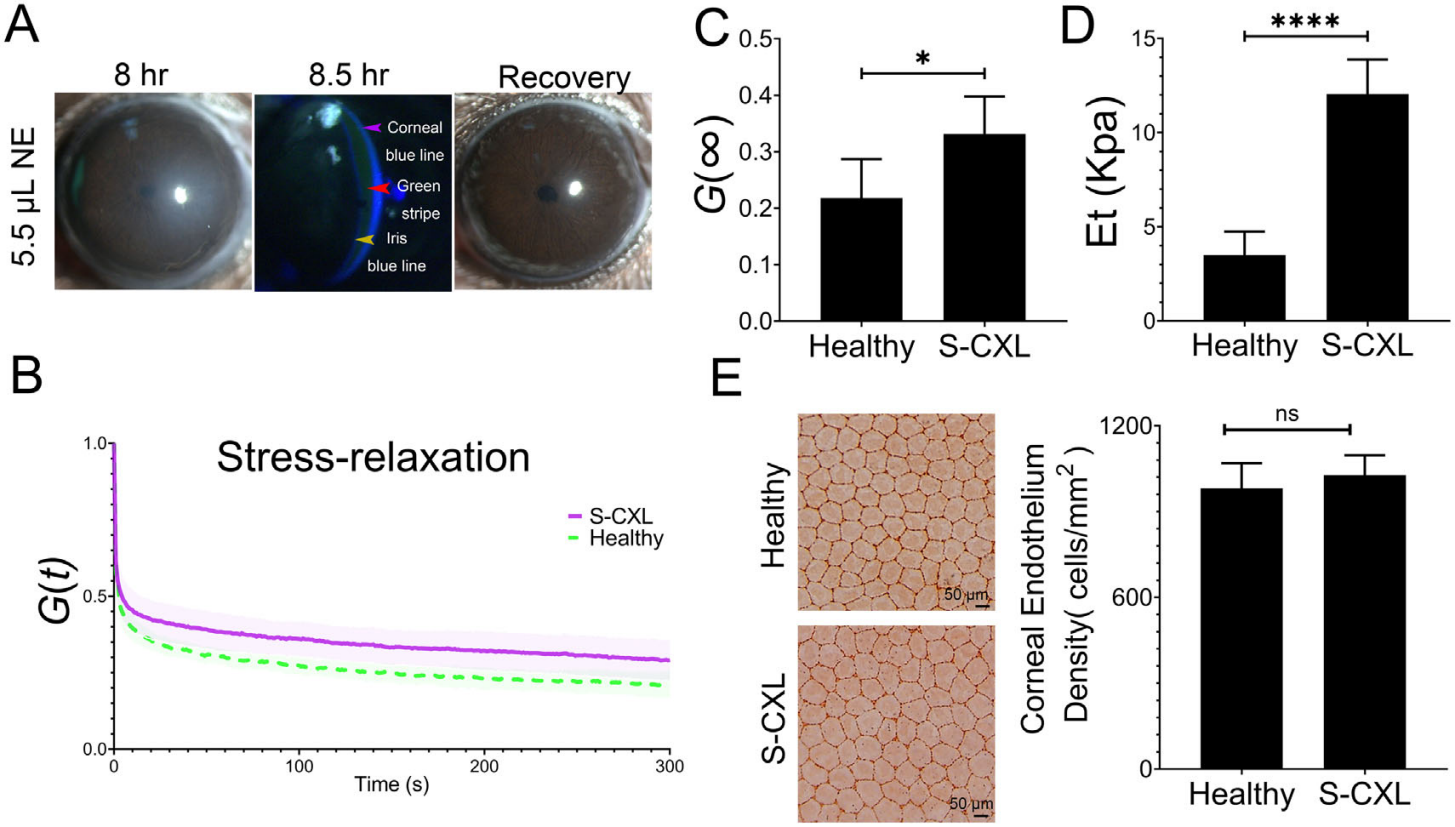
**High-dose NE was feasible for clinical CXL.** (**A**) Slit lamp images showed that the cornea displayed edema at 8 hours after 5.5 µL NE treatment (*left* photograph). The cornea could spontaneously recover to transparent status (*right* photograph). Riboflavin penetrated into the corneal stroma within 30 minutes according to the presence of both the weak green stripe (*red arrowhead*) and the visible iris blue line (*yellow arrowhead*). *Purple arrowhead*: Corneal blue line (*n* = 3). (**B**) Normalized stress-relaxation curves of the S-CXL and the healthy groups. The *line* stands for the average value and the *shadow* indicates the standard deviation (*n* = 5 and 6, respectively). (**C**) The S-CXL group had a larger normalized stress relaxation limit *G* (∞) than the healthy group (*n* = 5 and 6, respectively). *P < 0.05. (**D**) The S-CXL group had a larger elastic modulus than the healthy group (*n* = 5 and 6, respectively). ****P < 0.0001. (**E**) The S-CXL group had similar corneal endothelial cell shape and density compared with the healthy group, after the S-CXL group corneas were exposed to irradiation with 9 mW/cm^2^ UVA for 2.5 minutes (*n* = 5).

In the above, we tried riboflavin delivery at the transparent cornea state without edema, we think the cornea at the edema status might be easier for riboflavin infiltration to shorten the infiltration time (see Fig. 1A). Indeed, during clinical practice, the cornea with mild edema is acceptable for transepithelial CXL. Herein, to better fit the clinical scenario, we performed riboflavin infiltration at 8 hours after NE treatment and shortened riboflavin penetration time. Further, we adopted an optimized CLX protocol fit for mice by increasing the UVA energy intensity and decreasing the CXL duration (S-CXL). Then, 5.5 µL NE led to obvious epithelial junction dissociation at 8 hours (see Fig. 2C). Riboflavin was applied at 8 hours after NE treatment. A green stripe and a visible iris blue line could both be observed under the cobalt blue light illumination, indicating the riboflavin had diffused into the corneal stroma within 30 minutes (Fig. 5A). Moreover, the treated cornea could spontaneously recover to a healthy status (see Fig. 5A), indicating the high dose NE was still safe to use. After riboflavin penetration, we used an optimized CXL protocol. The velocity of stress relaxation of corneas in the S-CXL group was lower than the healthy group (Fig. 5B). Consistently, the value of *G* (∞) in the S-CXL group was larger than the healthy controls (Fig. 5C). There was also a significant increase in elastic modulus in the S-CXL group (Fig. 5D). All the above data suggested the S-CXL cornea possessed enhanced stiffness than the healthy cornea. To examine whether the penetrated riboflavin was enough to protect corneal endothelium during CXL, we stained the corneal endothelium from both groups. There was no difference in both the shape (Fig. 5E) and density (Fig. 5F) of endothelial cells between the two groups. The average endothelial density was 981.20 ± 88.47 cells/mm^2^ in the S-CXL group and 1027.80 ± 69.60 cells/mm^2^ in the healthy cornea group (see Fig. 5F). These results indicated this corneal crosslinking regimen in the S-CXL group effectively increased corneal biomechanics and protected the endothelium.

## Discussion

The corneal epithelium is the outermost barrier separating the environment and the intraocular tissues. In this study, we investigated the feasibility of using NE, a natural neurotransmitter existing in the cornea, as the enhancer to promote riboflavin penetration during CXL. To our knowledge, this is the first time NE was found to have the ability to overcome the epithelial barrier. Previously, we reported that NE could promote bacterial keratitis^24^^,^^25^ which was likely through the breakdown of epithelial integrity. The epithelial barrier is the major blockage during transepithelial CXL treatment. Therefore, facilitating riboflavin bypassing this epithelial barrier with little complication is the key for transepithelial drug development.

Various methods had been explored for this purpose. Chemical enhancers, such as tetracaine, benzalkonium chloride, ethylenediaminetetraacetic acid, and trometamol, were incorporated as supplementary medication in transepithelial protocols. However, they are all limited by the toxic side effects. For example, benzalkonium chloride, a widely applied chemical enhancer, has been reported to exert cytotoxic effects on corneal epithelium, ciliary epithelium, conjunctival, trabecular meshwork cells,^26^^–^^30^ and increased lymphocyte infiltration in ocular tissues.^26^ In addition, phonophoresis-assisted and iontophoresis-assistant techniques were developed for promoting riboflavin delivery.^31^^,^^32^ However, the rise in ocular surface temperature by 6 to 7°C after phonophoresis raised safety concerns.^33^ A 2-year clinical outcome of transepithelial CXL with iontophoresis showed a poor reduction in maximal keratometry (K_max_) than using the standard CXL protocol.^34^ There was a study showed that KC progression was blocked by transepithelial CXL with vitamin E-enhanced riboflavin solution.^35^ However, patients involved in this study were relatively milder cases. A novel hibiscus-like microsphere composite RF@ZIF-8 was developed as a carrier to promote riboflavin into the corneal stroma,^36^ which presented a better transepithelial CXL effect especially in avoiding the destruction of the cornea. At present, this composite has not been verified in patients with clinical KC. With all these efforts in riboflavin delivery, transepithelial drugs in clinical applications are still limited.

Because NE is a natural neurotransmitter detectable in the ocular surface, it seems safer to apply it to the ocular surface. Our study provided evidence that NE could be a potential candidate as a riboflavin enhancer, under both laboratory and clinical scenarios. However, the water-soluble property of NE makes it difficult to be delivered into the cornea as eye drops, as the corneal epithelium is hydrophobic. In this study, we used subconjunctival injection to deliver NE. The subconjunctival injection further limited the timely application of riboflavin. Several hours were needed for epithelial barrier dissociation after NE treatment, and riboflavin penetration requires extra time. The long duration of this protocol limits its application in clinics. Future efforts on NE modification could be encouraged, such as encapsulation of NE into liposomes or nanoparticles, to make the synchronous application of NE and riboflavin possible.

We also developed a novel protocol for laboratory corneal stiffness measurement. Regular indices to evaluate CXL efficacy for KC focused on K_max_, corrected distance visual acuity (CDVA), endothelial cell density, crosslinking line, and corneal biomechanics.^37^ Tensile test, inflation test, and indentation examination all can be performed to obtain the biomechanical properties of cornea in vitro.^22^^,^^38^^–^^41^ Mouse cornea had a relatively small size, therefore, commercially available extensometers for tensile tests could not fit the mouse cornea.^22^^,^^38^ In contrast, the indentation test offered a convenient approach without the need for an additional customized holder. In this study, we tested the mechanical properties of the stromal layer by freeze-slicing the cornea and positioning the indenter on a target area of the tissue.^42^ The operation was relatively simple, and the target area of the stroma could be easily located without scraping the epithelium and physically separating the stroma tissue.^30^^,^^43^

Collectively, our study suggests that NE is safe and efficient in promoting riboflavin infiltration under both the laboratory and clinical CXL settings, exhibiting potential for clinical use during transepithelial crosslinking.

## Supplementary Material

Supplement 1
