# Supplementary material for: Norepinephrine as an Enhancer Promoting Corneal Penetration of Riboflavin for Transepithelial Corneal Crosslinking
Source: Transl Vis Sci Technol. 2023 Feb 14;12(2):21. doi: 10.1167/tvst.12.2.21 (PMC9932548; doi:10.1167/tvst.12.2.21)
Supplement: Supplement 1 [file tvst-12-2-21_s001.pdf]

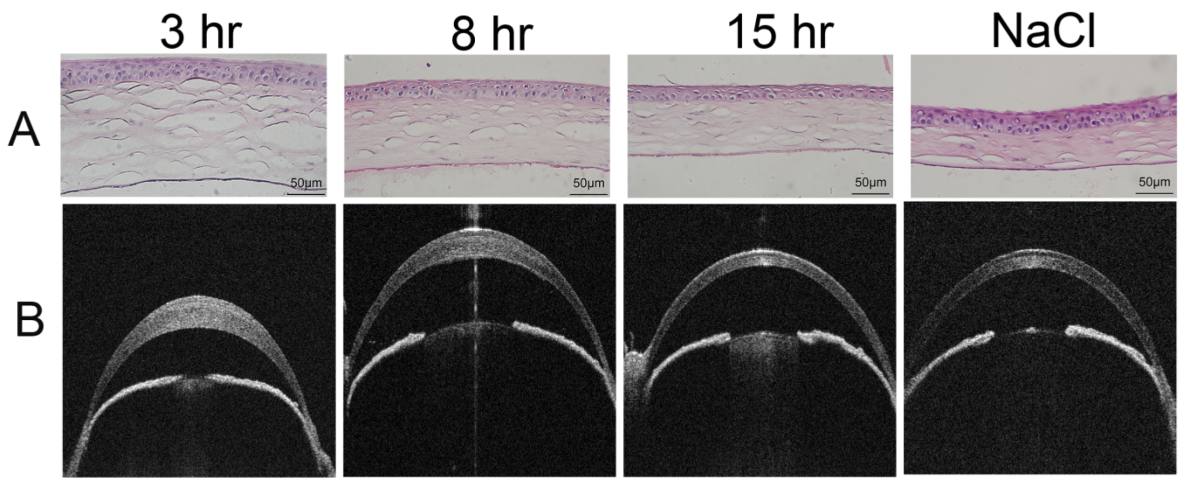

Supplementary Figure 1. Corneal edema and recovery at different time points after NE treatment. (A) Histological examinations of corneas with H&E staining indicated corneal edema could be observed between 3 hrs and 8 hrs after NE treatment. The treated cornea recovered at 15 hr after NE treatment. *n* = 4. (B) OCT examinations indicated that the corneal thickness increased significantly at 3 hr and 8 hr. Corneas recovered to normal thickness at 15 hr after NE treatment. *n* = 4.

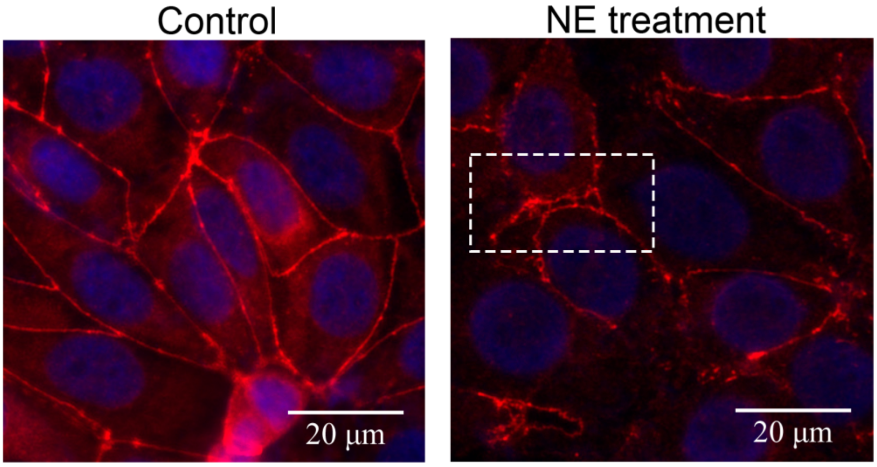

Supplementary Figure 2. *In vitro* immunostaining showed corneal epithelial junction dissociation after NE treatment. Corneal epithelial cells treated with 10 μM NE for 1 hr displayed dissociation of ZO-1 (dotted box) compared with control subjects. $n = 3$ times of experiments.

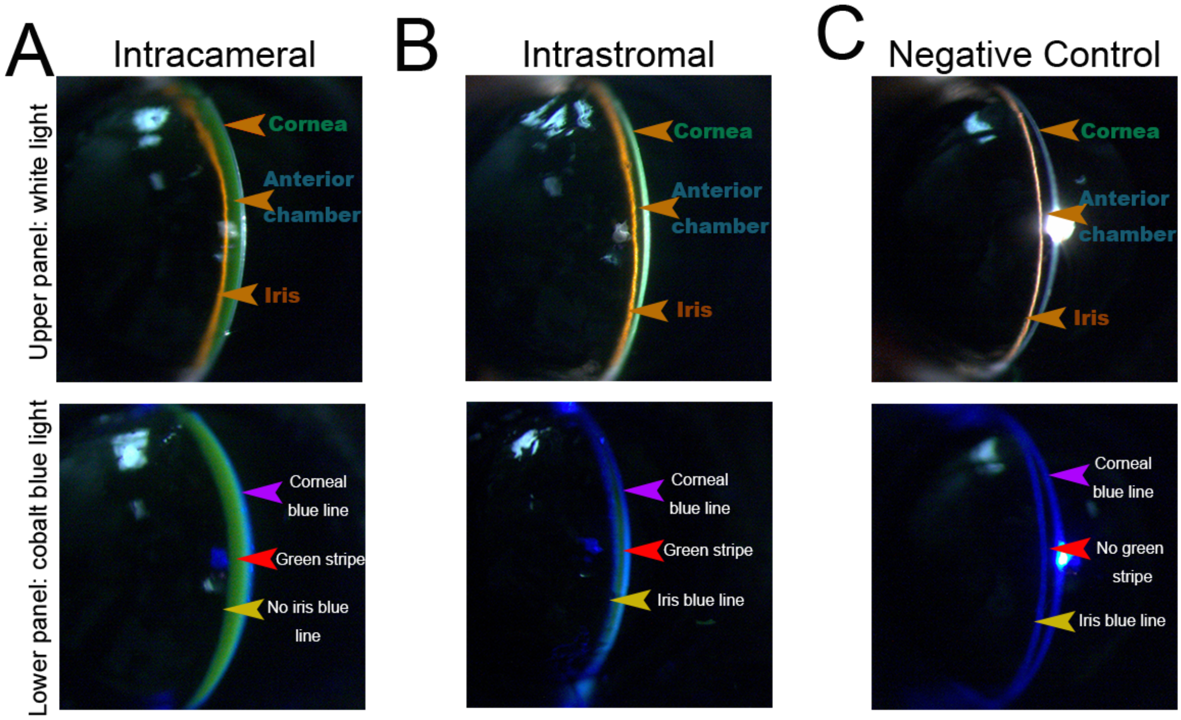

Supplementary Figure 3. Positive controls for indicating riboflavin penetration. Upper panel: white light images. Lower panel: cobalt blue light images. (A) Diffused green fluorescence could be observed between the iris and the cornea under white light illumination by the slit lamp after intracameral injection of riboflavin. An intense green stripe (red arrowhead) without iris blue line (yellow arrowhead) was visible under cobalt blue light. Purple arrowhead: corneal blue line. $n = 3$. (B) Green fluorescence is mainly concentrated inside the cornea after intrastromal injection of riboflavin under white light illumination. Both the green stripe with weak intensity (red arrowhead) and the iris blue line (yellow arrowhead) were visible under the cobalt blue light illumination. Purple arrowhead: corneal blue line. $n = 3$. (C) No green stripe (red arrowhead) was observed under the cobalt blue light when no riboflavin was injected. $n = 3$.

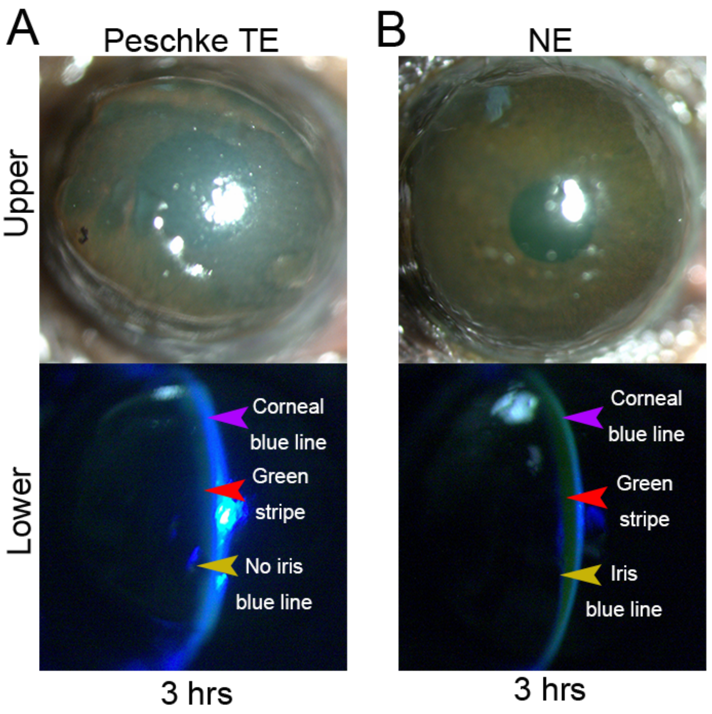

Supplementary Figure 4. Prolonged application of Peschke TE caused corneal damage. Upper panel: white light images. Lower panel: cobalt blue light images. (A) Representative images of slit lamp microscopy of corneas applying Peschke TE for 3 hrs. n = 3. (B) Representative images of slit lamp microscopy of corneas after 3 hrs riboflavin penetration in the 2 μL NE treatment group for 15 hrs. n = 3.

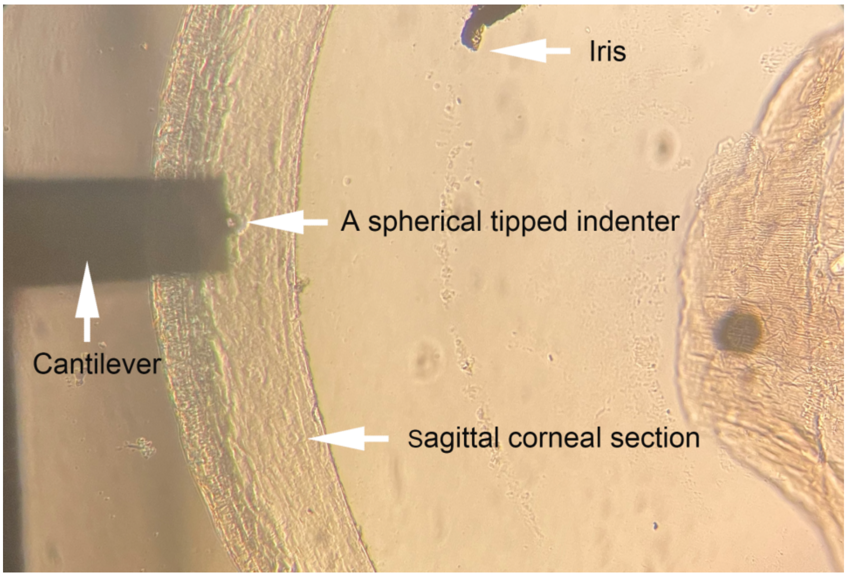

Supplementary Figure 5. Presentation of the indentation test. A spherical tipped indenter with a radius of 24.5 μm and a cantilever with a stiffness of 0.59 N/m were used for all indentation tests. Indentation points located in the middle of each sagittal corneal section were loaded.
